# Hyaluronate-coated novasomes as a nanoplatform for targeted delivery of *Beta vulgaris* subsp. *cicla* extract: formulation, characterization, and phytochemical profiling

**DOI:** 10.1038/s41598-026-56921-8

**Published:** 2026-06-16

**Authors:** Doaa A. H. Deabes, Eman A.W. El-abd, Alaa M. Saleh, Eman S. Shalaby

**Affiliations:** 1 Pharmacognosy Department, National Research Centre, 33 El Buhouth St, Dokki, P.O. Box 12622, Giza, Egypt; 2 Department of Chemistry of Natural and Microbial Products, Pharmaceutical and Drug Industries Research Institute, National Research Centre, 33 El Buhouth St, Dokki, P.O. Box 12622, Giza, Egypt; 3 Pharmaceutical Technology Department, National Research Centre, 33 El Buhouth St, Dokki , P.O. Box 12622, Giza, Egypt

**Keywords:** Antimicrobial, Antioxidant, GC-MS, LC-MS/MS, Novasomes, Biochemistry, Biotechnology, Chemistry, Drug discovery, Microbiology, Plant sciences

## Abstract

Hyaluronate-coated novasomes as an eco-innovative delivery system to enhance the antimicrobial and antioxidant efficacy of *Beta vulgaris* subsp. *cicla* (BV) aerial parts extracts were developed and characterised in this study. The phytochemical profile was established using GC-MS and LC-MS/MS, along with the isolation of key biomarkers. Two novasome formulations (BV1 and BV2) were synthesized, and the optimized BV1 was subjected to comprehensive characterization, including encapsulation efficiency (EE), particle size, zeta potential, FTIR, and TEM. The biological activities were evaluated via DPPH radical scavenging and agar well assays against *Staphylococcus aureus*, *Escherichia coli*, and *Candida albicans*. GC-MS analysis of the dichloromethane fraction identified 15 compounds (89.64%), dominated by fatty acids: oleic acid (30.52%), palmitic acid (21.92%), and stearic acid (10.50%). LC-MS/MS analysis of the ethanolic extract revealed 33 metabolites, primarily flavonoids and phenolic acids. The isolated compounds included oleanolic acid, campesterol, isovitexin, and apigenin-*O*-hexoside. While the crude extract exhibited limited antimicrobial activity (effective only against *C. albicans*), encapsulation within hyaluronate-coated novasomes significantly enhanced its biopotency. BV1 exhibited some inhibitory trends against all tested microorganisms, whereas BV2 was effective only against bacterial strains. Regarding radical scavenging activity, BV1 exhibited significantly higher antioxidant activity (IC_50_ 33.4 µL/mL) than the crude extract (IC_50_ 55.2 µL/mL**)** and BV2 (IC_50_ 82.6 µL/mL). The results demonstrate that hyaluronate-coated novasomes serve as a superior delivery vehicle, expanding the limited antimicrobial profile of *Beta vulgaris* extract and preserving its antioxidant capacity.

## Introduction

Global health challenges are increasingly defined by the dual threat of microbial resistance and oxidative stress-induced chronic diseases. The rise of multidrug-resistant pathogens and the limitations of synthetic antioxidants have shifted the scientific focus towards natural alternatives found in plant secondary metabolites. Plants serve as advanced biofactories for secondary metabolites, notably flavonoids and anthocyanins, which are essential for plant defence and human therapeutic uses^1^. Among these, the *Chenopodiaceae* family, specifically *Beta vulgaris* subsp. *cicla* (Swiss Chard) (BV), has emerged as a significant source of bioactive compounds^2^^,3^.

Despite being a nutrient-dense vegetable rich in betalains, flavonoids (such as vitexin and vicenin II), and saponins, the therapeutic potential of *B. vulgaris*is frequently compromised^4^^,5^, Many of its most potent constituents, including oleic and oleanolic acids, are highly lipophilic, leading to poor aqueous solubility and low bioavailability at the target sites^6^^,7^^,8^. This creates a “delivery gap” where the inherent biopotency of the extract is lost during administration^9^^,10^.

Advanced nano-vesicular systems, specifically novasomes, offer a promising solution to overcome these pharmacokinetic barriers. Unlike traditional liposomes, novasomes are multi-bilayered vesicles composed of cholesterol, free fatty acids, and polyoxyethylene fatty acid monoesters, providing a high-capacity central core for lipophilic drug entrapment^11^. However, for topical or targeted applications, the surface characteristics of these vesicles are crucial.

By incorporating hyaluronic acid (HA), a natural, biocompatible, and anionic glycosaminoglycan, the nanovesicles can be functionally modified. HA not only improves the “stealth” qualities and stability of the particles but also possesses intrinsic bacteriostatic properties and a high affinity for cellular uptake^12–14^.

Therefore, the present study addresses the challenge of delivering *Beta vulgaris* subsp. *cicla* extract by developing hyaluronate-coated novasomes. By encapsulating the aerial parts’ extract into these eco-innovative nanocarriers, this work aims to optimize the stability, diffusion, and biological performance of the plant’s metabolites, probably providing an enhanced antioxidant and antimicrobial strategy for sustainable skin health solutions. The novelty of this work lies in the dual-functional design of the carrier: the novasome core provides a stable environment for sensitive betalains, while the hyaluronic acid coating serves as a targeting and stabilizing agent. This study provides a unique comparative analysis between free and nano-encapsulated extracts, offering new insights into how hyaluronate-functionalized nanocarriers can modulate the antimicrobial spectrum and antioxidant efficiency of traditional herbal extracts.

## Results and discussion

### Phytochemical investigation

#### Gas chromatography–mass spectrometry (GC-MS) identified metabolites of the dichloromethane fraction of aerial parts of *Beta vulgaris*subsp*. cicla.*

GC-MS analysis of the dichloromethane fraction of aerial parts of *Beta vulgaris* subsp. *cicla* represented in Table 1 revealed the identification of fifteen oxygenated compounds representing 89.64%. The most abundant compounds were oleic acid (30.52%), which is *a mono-unsaturated omega-9 fatty acid*, followed by the saturated fatty acid palmitic acid (21.92%), which was previously identified in Swiss chard^15^. Stearic acid (10.50%) and triterpenes and sterols were also identified in the plant (Ursane-3,12-diol, Cholesteryl benzoate, Stigmast-5-en-3-ol-oleate, R1-Barrigenol and Olean-12-ene-3,15,16,21,22,28-hexol).

**Table 1:** Identified compounds by GC-MS analysis of the dichloromethane fraction of aerial parts of* Beta vulgaris *subsp*. cicla.*

No	Name	RRt	M.WT	M.F	Relative Area %
1	Palmitic acid	0.89	256	C₁₆H₃₂O₂	21.92
3	Elaidic acid, methyl ester	0.97	296	C_19_H_36_O_2_	5.38
4	Nonanoic acid, 9-(o-propylphenyl)-, methyl ester	0.98	290	C_19_H_30_O_2_	5.51
5	Methyl stearate	0.99	298	C_19_H_38_O_2_	5.25
6	Oleic Acid	1	282	C_18_H_34_O_2_,	30.52
8	Stearic acid	1.01	284	C_18_H_36_O_2_,	10.50
9	6,9-Octadecadienoic acid, methyl ester	1.02	294	C_19_H_34_O_2_	3.80
10	Oxiraneundecanoic acid, 3-pentyl-, methyl ester	1.03	312	C_19_H_36_O_3_	1.85
11	Ursane-3,12-diol	1.24	444	C_30_H_52_O_2_	1.61
12	Cholesteryl benzoate	1.35	490	C_34_H_50_O_2_	0.66
13	Stigmast-5-en-3-ol oleate	1.43	678	C_47_H_82_O_2_	1.04
14	R1-Barrigenol	1.52	506	C_30_H_50_O_6_	0.65
15	Olean-12-ene-3,15,16,21,22,28-hexol	1.62	506	C_30_H_50_O_6_	0.95
Total identified compounds					89.64

RRt.: relative retention time relative to oleic acid M.F.: molecular formulaM.wt.: molecular weight

#### Metabolites identified by Liquid chromatography–tandem mass spectrometry (LC-MS/MS) of aerial parts of *Beta vulgaris*subsp*. cicla.*

The crude ethanol extract of *Beta vulgaris* subsp. *cicla* aerial parts was subjected to LC-MS/MS under the previous condition, a commonly used technique for the identification of different metabolites. Metabolites were assigned based on comparing the retention time (Rt), MS data highly accurate mass, molecular ion fragmentation pattern, and the predicted formulas data in the literature and databases listed in the authorized websites, PubChem and Mass Bank of North America (MoNA), together with the database (Peakview software program). The ionization of the compounds in our study was performed in the negative ionization mode. The identified compounds, their mass fragments, and molecular formulas are listed in Table 2. Annotations of different chromatographic peaks allowed the identification of 33 compounds belonging to various metabolite classes, including flavonoids, phenolic acids, and their derivatives, which represented the major compounds. In addition, coumarin, fatty acid, and amino acid were identified.

**Table 2:** Metabolites identified by LC-MS/MS of aerial parts of *Beta vulgaris *subsp.* cicla *in negative ionization mode.

No.	Identification	Rt.	Parent ion (M-H)	Production	M.F.	Chemical class	References
1	Dihydroferulic acid	1.31	195	177 (M-H-H₂O), 162 (M-H-CO₂-CH₃), 136 (M-H-H₂O-CH₃)	C_10_H_12_O_4_	Phenolic acid	^16^
2	Ferulic acid	1.35	193	178 (M-H-CH₃), 149 (M-HCOO), 134 (M-H-CH₃-CO₂)	C_10_H_10_O_4_	Phenolic acid	^16^
3	Sinapaldahyde	2.28	207	192 (M-H-CH₃), 177 (M-H-2CH₃), 133	C_11_H_12_O_4_	Phenolic acid	^16^
4	Protocatechuic acid	3.10	153	135 [M-H-H₂O], 109 [M-H-CO₂], 112	C_7_H_6_O_4_	Phenolic acid	^17^
5	P-Coumaric acid	4.09	163	145 [M-H₂O], 119 [M-H-CO₂]	C_9_H_8_O_3_	Phenolic acid	^18^
6	Vanillic acid	4.27	167	152(M-H-CH₃), 123(M-H-CO₂), and 108(M-H-CH₃-CO₂)	C_8_H_8_O_4_	Phenolic acid	^16^
7	*p*-Coumaric acid hexose	5.26	325	207, 163,145, 119	C_15_H_18_O_8_	Phenolic acid Derivative	^19^
8	Isovitexin 2’’-O-rhamnoside	5.30	577	341 (M-H-90–146), 323 (M-H-90–146-18), 293	C_27_H_30_O_14_	Flavonoid	^16^
9	Isovitexin (Apigenin 6-*C* glucoside)	5.49	431	413,341,311,283	C_21_H_20_O_10_	Flavonoid *C*-glycoside	^16^
10	apigenin-C pentoside – C-hexoside	5.54	563	413,293,311	C_26_H_28_O_14_	Flavonoid	^10^
11	Vitexin	6.28	431	341,311,283	C_21_H_20_O_10_	Flavonoid C-glycoside	^16^
12	Apigenin-*O-*hexoside	6.28	431	269 [M-H-hexoside], 227	C_21_H_20_O_10_	Flavonoid	^20^
13	Quercetin-*O*-hexoside	6.35	478	301 [M-H-GLU-CH_3_], 271 [M-H-GLU-CH_3_- CH₂O], 255 [M-H-GLU-CH_3−_CO-H₂O]	C_21_H_20_O_12_	Flavonoid	^21^
14	Rutin	6.40	609	301 [M-H-rutinoside], 271 [M-H-rutinoside-CH₂O], 255 [M-H-rutinoside-CO-H₂O], 179, 151	C_27_H_30_O_16_	Flavonoid	^16^
15	Diosmetin 8-C-glucoside-6-C-pentoside	6.54	593	473 (M-H-120), 429 (M-H-120-CO_2_), 413 (M-H-120-60), 298, 297	C_37_H_30_O_15_	Flavonoid	^16^
16	Vitexin-2’-*O*-rhamnoside	6.86	577	457(M-H-120), 311(M-H-120–146), 293	C_27_H_30_O_14_	Flavonoid	^16^
17	Kaempferol-*O*-rutinoside	6.90	593	285 [M-H-rutinoside], 255 [M-H-rutinoside-CH₂O], 227 [M-H-rutinoside-2CHO]	C_27_H_30_O_15_	Flavonoid	^16^
18	Luteolin-8-C-β-D-glucopyranoside-7-*O*-rhamnoside	6.97	593	473 (M-H-120), 327 (M-H-120–146), 298	C_27_H_30_O_15_	Flavonoid	^22^
19	Kaempferol 7-*O*-hexoside	7.16	447	285, 255, 227	C_21_H_20_O_11_	Flavonoid O-glycoside	^16^
20	Luteolin-*O*-hexoside	7.16	447	401,285, 267, and 255.	C_21_H_20_O_11_	Flavonoid	^16^
21	Apigenin	8.17	269	251,225,197,149	C_15_H_10_O_5_	Flavonoid	^16^
22	Trihydroxy octadecadienoic acid	8.35	327	309,229,211,171	C_18_H_32_O_5_	Fatty acid	^16^
23	Epicatechin	8.51	289	245(M-H-CO₂).	C_15_H_14_O_6_	Flavonoid	^16^
24	Quercetin	9.46	301	273, 255, 151, 151, 151, 151, 151, 151,179	C_15_H_10_O_7_	Flavonoid	^23^
25	Dicaffeoylquinic acid methyl ester isomer	10.33	529	367, 353, 193	C_26_H_26_O_12_	Phenolic acid derivatives	^24^
26	Proline-betaxanthin	10.67	309	283, 195	C_14_H_16_N_2_O_6_	Betaxanthin derivatives	^25^
27	Chrysoeriol	10.71	299	284(M-H-CH₃), 137	C_16_H_12_O_6_	Flavonoid	^16^
28	Limocitrin	11.42	345	330,315,276	C_17_H_14_O_8_	Flavonoid	^16^
29	Grevilline B	12.57	339	321,307,295,265	C_18_H_12_O_7_	Catechols	^26^
30	Syringic acid	13.11	197	182 (M-H-CH₃), 179 (M-H-H₂O), 153 (M-H-CO₂), 129, 113, 85	C_9_H_10_O_5_	Phenolic acid	^16^
31	Esculin (6,7-dihydroxycoumarin 6-glucoside)	12.75	339	321,293,177	C_15_H_16_O_9_	Coumarin glycoside	^16^
32	Glutamine-betaxanthin	13.33	340	321, 277	C_14_H_17_N_3_O_7_	Amino acid	^27^
33	Chrysin (5,7-dihydroxyflavone)	20.22	253	235 (M-H-CH₃), 209	C_15_H_10_O_4_	Flavonoid	^16^

Rt: retention time, M.F: molecular formula.

#### Isolation and identification of the major compounds from the dichloromethane fraction (Fig. 1)

The dichloromethane extract was loaded repeatedly to TLC chromatography; then, the collected bands were examined under UV at λ max 254 and 365 nm. The bands were further purified several times, resulting in the isolation of two compounds (1–2) that showed positive Salkowski’s results and attained different colours with 10% sulphuric acid as a spraying reagent.

 Compound 1: (Oleanolic acid)

White crystals soluble in chloroform (Rf = 0.54).

The ¹H-NMR spectrum of oleanolic acid shows numerous methyl groups at 0.80, 0.85, 0.90, 0.92, 0.92, 0.92, 0.92, 0.98, 1.10, and 1.18 and a characteristic olefinic proton of the C12–C13 double-bond pentacyclic triterpenoid at 5.40 (1 H, brs, H-12). This suggests the presence of an olea-12-ene skeleton. One methyl proton at 3.34 (1 H, t, J = 8.2 Hz, 3-H) is compatible with at least one hydroxyl group on the oleanolic acid olean-12-ene skeleton. On the other hand, the ¹³C-NMR spectrum displays signals related to an oxygenated.

carbon signal at 78.88 (C-3), one tri-substituted double bond at 122.18 (C-12), 144.45 (C-13), and one carboxyl group at 180.64 (C-28). Furthermore, ¹³C-NMR signals from C-18 to C-22 at 41.68 (C-18), 46.35 (C-19), 30.52 (C-20), 32.88 (C-21) and 31.55 (C-22) indicate that oleanolic acid derives from the oleanyl carbocation. The MS spectra for OA obtained by LC-ESI-ITTOF found that the five most prominent peaks corresponded to the m/z molecular fragments 457, 203, 191, 218, 75, 29, and 30.

 Compound 2: (Campesterol)

It had a melting point of 160–164 °C and was obtained in the form of a white powder. EI-MS showed characteristic peaks at an m/z of 400, with molecular ion and fragment ion m/z values of 382, 367, 273 and 255 indicating a molecular formula of C₂₈H₄₈O. The ¹H-NMR spectra data was almost identical to that of campesterol in the literature, with ¹H-NMR displaying dH for one olefinic methine proton (dH 5.46 H-6), one hydroxyl proton (dH 4.49 OH), six methyl protons (dH 0.76 H-18, 0.63 H-19, 0.76 H-21, 0.73 C-26, 0.79 H-27, and 0.62 C-28), ten methylene protons (dH 1.94 H-1, 1.78 H-2, 1.56 H-4, 1.10 H-7, 1.09 H-11, 1.16 H-12, 2.09 H-15, 1.86 H-16, 2.15 H-22, and 1.04 H-23), and eight methine protons (dH 3.48 H-3, 1.76 H-8, 0.95 H-9, 1.42 H-14, 1.75 H-17, 2.23 H-20, 0.85 H-24, and 1.22 H-25).

The ^13^C NMR and DEPT revealed 28 carbon signals for six methyl carbons (δC 15.54 C-18, 12.14 C-19, 14.22 C-21, 21.23 C-26, 19.88 C-27, and 15.54 C-28), ten methylene carbons (δC 37.40 C-1, 31.79 C-2, 42.43 C-4, 32.08 C-7, 23.18 C-11, 39.88 C-12, 24.42 C-15, 26.18 C-16, 34.16 C-22, and 34.10 C-23), eight methine carbons (δC 70.01 C-3, 31.06 C-8, 49.42 C-9, 54.93 C-14, 53.10 C-17, 35.30 C-20, 38.21 C-24, and 31.86 C-25), three quaternary carbons (δC 143.41 C-5, 35.66 C-10, and 43.95 C-13), and one olefinic methine carbon (δC 121.92 C-6). Compound 1 was identified as campesterol in accordance with the spectral data that are stated in the literature. ³¹.

####  Compounds isolated from ethanol extract (Fig. 1)

 Compound 3: (Isovitexin)

Yellow powder soluble in methanol, Rf. 0.73 In the system (CH₂Cl₂: MeOH 9:1), UV λ _max_ in methanol is 334,270, which indicates the flavone class; sodium methoxide 395,327,280 reveals the presence of a free 4^’^ OH; HCl/ALCl₃ 390,344,304,278 shows that there is no ortho dihydroxy group; and NaOAC/boric acid 344,304,272. ^1^H NMR (400 MHz, MeOH): 7.96 (2 H, d, J = 8.6, 2’,6’H), 6.92 (2 H, d, J = 8.6, 3’,5’H), 6.60 (1H, s, 8-H), 6.26 (1H, s, 3-H), 5.00 (1H, d, J = 9.92 H1‶) C-sugar, and 4.84 (1H, s). TOF/MS-ESI: 431 (M-H),413(M-H-H_2_O),341(M-H-90),311(M-H-120). From ¹H-NMR^,^ ESI-MS spectral data and by comparison to published data, compound 4 could be identified as isovitexin. It was also identified by the UPLC/MS/MS peak (9). Isovitexin has also been shown to have antioxidant, anti-inflammatory, anti-Alzheimer’s, and antiviral properties similar to those of its isomer, vitexin^28^.

 Compound 4: (Apigenin-7-***O***-β-D-glucoside)

A white powder soluble in methanol compounds (5, flavone) was identified as a flavonoid glucoside. UV showed a bathochromic shift with NaOMe in band I, indicating the presence of a hydroxyl group at C-4’, but no bathochromic shift with NaOAc/H₃BO₃ indicated the absence of a hydroxyl group at C-3’ and an occupied hydroxyl group at C-7. The presence of a singlet proton at δ 6.92 ppm (H-3) confirmed the flavone nature of compound 5. Signals at δ 5.01 and δ 5.13 ppm (H-1’’) and δ 3.5–4.15 and δ 3.22–3.91 ppm (H-2’’, 3’’, 4’’, 5’’, and 6’’) confirmed the presence of β-D glucose at C-7 for compoud 4.^13^C-NMR showed anomeric carbons at 99.13 and 103.19 (C-1’’). The compound 5 was confirmed to be apigenin-7-O-β-D-glucoside. That is confirmed by the UPLC/MS/MS peak (1233 ESI/MS: 431,269,227). 

**Fig. 1:**
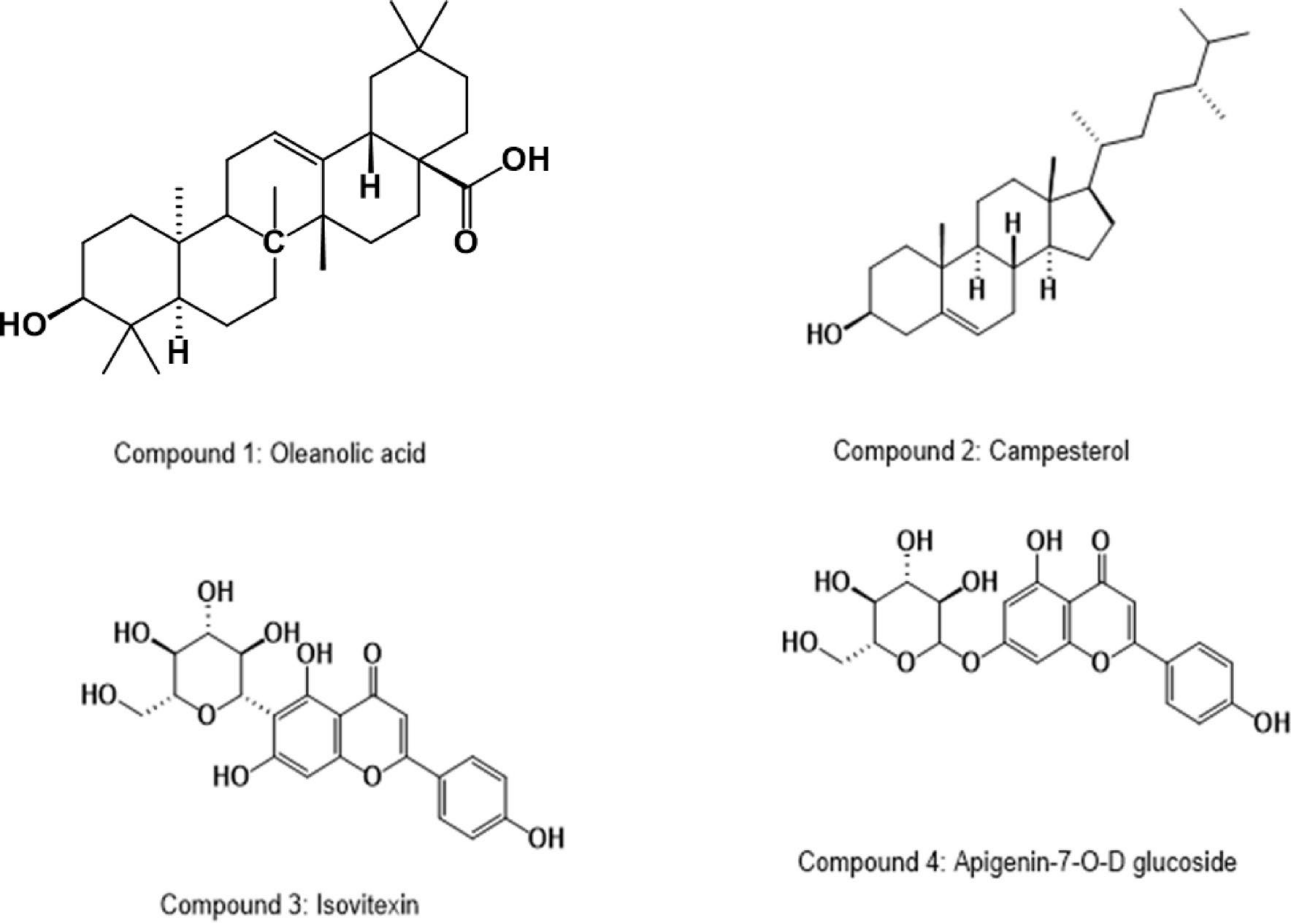
Structures of major compounds isolated from dichloromethane fraction and total ethanol extract from *Beta vulgaris* subsp.* cicla *aerial parts

### Biological activities

#### Antimicrobial activity

**Fig. 2:**
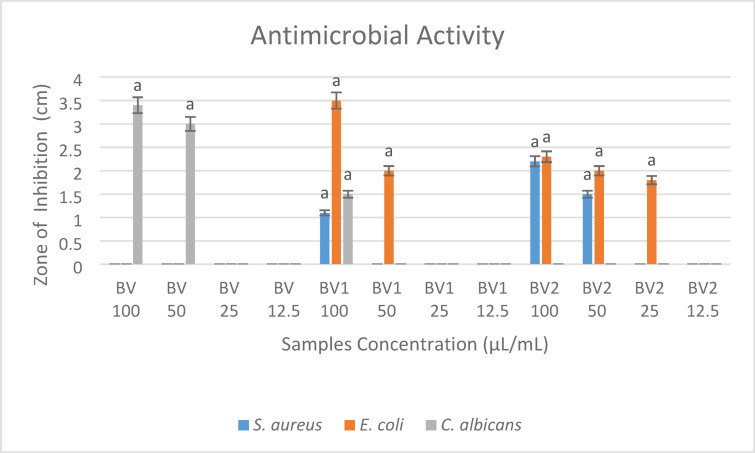
Antimicrobial activity of different concentrations of BV and its nano-formulations. Data are presented as mean±SE, (n=3). Statistical significance was determined determined by One-way ANOVA followed by Tukey’s post-hoc test.

The antimicrobial activity of *Beta vulgaris* crude extract (BV) and its novasome formulations (BV1 and BV2) was evaluated against *S. aureus*,* E. coli*, and *C. albicans* (Table 3; Fig. 2). The free extract (BV) showed no inhibitory effect against the bacterial strains at the tested concentrations (up to 1000 mL/mL). Conversely, the novasome formulations exhibited observable, though modest, zones of inhibition. However, statistical analysis revealed that these differences were not statistically significant (*P* > 0.05), likely due to the high standard deviations common in the well-diffusion assays of crude plant extracts. BV1 produced a zone of 3.5 ± 2.1 cm against *E. coli* at 1000 mL/mL, while BV2 demonstrated inhibitory trends against both *S. aureus* (2.2 ± 1.08 cm) and *E. coli* (2.3 ± 2.0 cm).

**Table 3:** Antimicrobial activity of *Beta vulgaris* and its nano-formulas.

Sample	Concentration (µL/mL)	*S. aureus*	*E. coli*	*C. albicans*
BV	1000	0 ± 0^a^	0 ± 0^a^	3.4 ± 2.3^a^
	500	0 ± 0^a^	0 ± 0^a^	3.0 ± 2.8^a^
	250	0 ± 0^a^	0 ± 0^a^	0 ± 0^a^
	125	0 ± 0^a^	0 ± 0^a^	0 ± 0^a^
BV1	1000	1.1 ± 2^a^	3.5 ± 2.1^a^	1.5 ± 1.9^a^
	500	0 ± 0^a^	2.0 ± 1.8^a^	0 ± 0^a^
	250	0 ± 0^a^	0 ± 0^a^	0 ± 0^a^
	125	0 ± 0^a^	0 ± 0^a^	0 ± 0^a^
BV2	1000	2.2 ± 1.08^a^	2.3 ± 2.0^a^	0 ± 0^a^
	500	1.5 ± 2.0^a^	2.0 ± 2.5^a^	0 ± 0^a^
	250	0 ± 0^a^	1.8 ± 2.3^a^	0 ± 0^a^
	125	0 ± 0^a^	0 ± 0^a^	0 ± 0^a^
DMSO	1000	0	0	0
Antibiotic		4.5 ± 2.5	5.0 ± 1.79	3.0 ± 2.4

Data are presented as mean ± SD, (n=3). Significance of differences was tested according to One-way ANOVA followed by Tukey’s post-hoc test.

#### Antioxidant activity

**Fig. 3:**
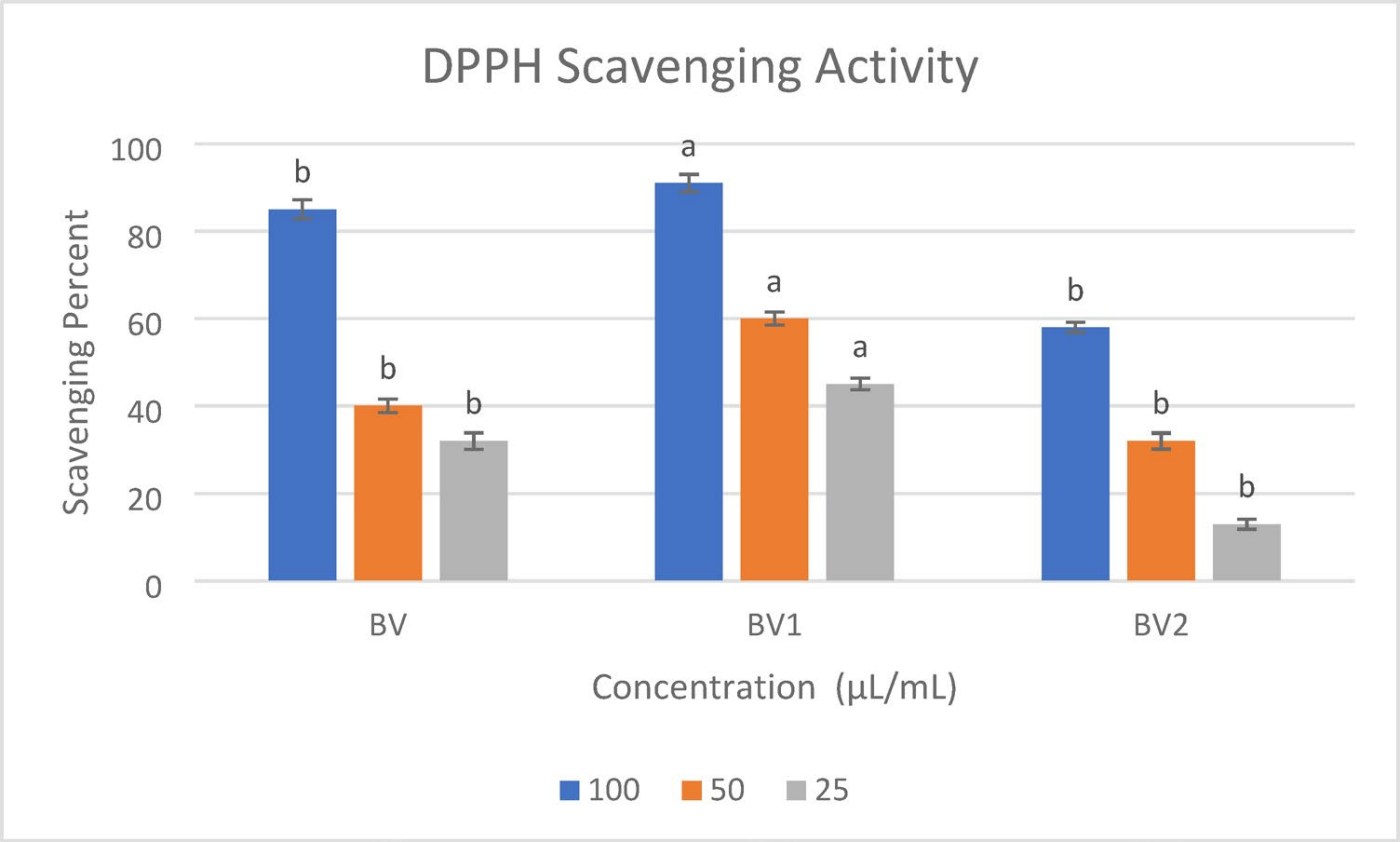
Graph representing the percent inhibition of DPPH versus samples. Data are presented as mean±SE, (n = 3). Statistical significance was determined determined by One-way ANOVA followed by Tukey’s post-hoc test.

The antioxidant activity of *Beta vulgaris* crude extract (BV) and its novasome formulations (BV1 and BV2) exhibited a clear concentration-dependent scavenging effect against the DPPH free radical. As shown in Table 4; Fig. 3, the encapsulation of the extract within novasomes significantly modulated its antioxidant capacity.

The IC_50_ values revealed that the encapsulation process significantly influenced the radical scavenging efficiency. BV1 was identified as the most potent antioxidant with a significantly lower IC_50_ of 22.48 mg/mL (*P* < 0.05) compared to the free extract (BV), which exhibited an IC_50_ of 30.05 mg/mL. While the BV2 formulation initially appeared significantly less potent in volumetric units, its mass-standardised IC_50_ (27.59 mg/mL) was found to be statistically comparable to the free extract (*P* > 0.05), though still significantly less effective than BV1. These results demonstrate that the BV1 formulation provides the highest antioxidant yield per unit mass of the delivery system.

**Table 4:** Antioxidant activity of *Beta vulgaris* and its nano-formulas against DPPH.

Sample	Concentration (µL/mL)	% DPPH Inhibition
BV	100	85.0 ± 2.2^b^
	50	40.0 ± 1.5 b
	25	32.0 ± 1.9^b^
	IC_50_	30.05 mg/mL
BV1	100	91.0 ± 2.0^a^
	50	60.0 ± 1.5^a^
	25	45.0 ± 1.4^a^
	IC_50_	22.48 (mg/mL)
BV2	100	58.0 ± 1.2^b^
	50	32.0 ± 1.8^b^
	25	13.0 ± 1.2^b^
	IC_50_	27.59 (mg/mL)

Data are presented as mean ± SD, (n = 3). Significance of differences (P < 0.05) was tested according to One-way ANOVA followed by Tukey’s post-hoc test.

### Characterization of the selected hyaluronate-coated novasomes

#### Extract content % measurements, particle size, zeta potential, and polydispersity index (PDI)

A selection has been made of the most effective antioxidant formulation to be further characterized. The amount of extract retained within selected coated novasomes is referred to as ‘drug content’. The drug content of the selected system was 62.03% (Table 5). All the results clearly indicated that the novasomes stabilized hyaluronate and prepared with mixed SAA and FAA at a percentage of 2:1 of span 60: oleic acid could effectively nanoencapsulate the extract after preparation.

The results of zeta potential, particle size, and PDI of the prepared novasomes are presented in Table 5. Particle size value was in the nanorange (340.2 nm). The value of the zeta potential often indicates how stable the nanoparticles are^29^. The zeta potential value was negative. This could be due to the anionic nature of sodium hyaluronate. The selected formulation zeta potential values are higher than 30, suggesting the high stability of the prepared systems. The PDI value was below 0.5, indicating narrow size distribution. So, Formulation (BV1) showed high EE, high zeta potential, low PDI values, and reasonable particle size, suggesting it as a successful system to encapsulate the extract.

#### Transmission electron microscopy (TEM)

**Fig. 4:**
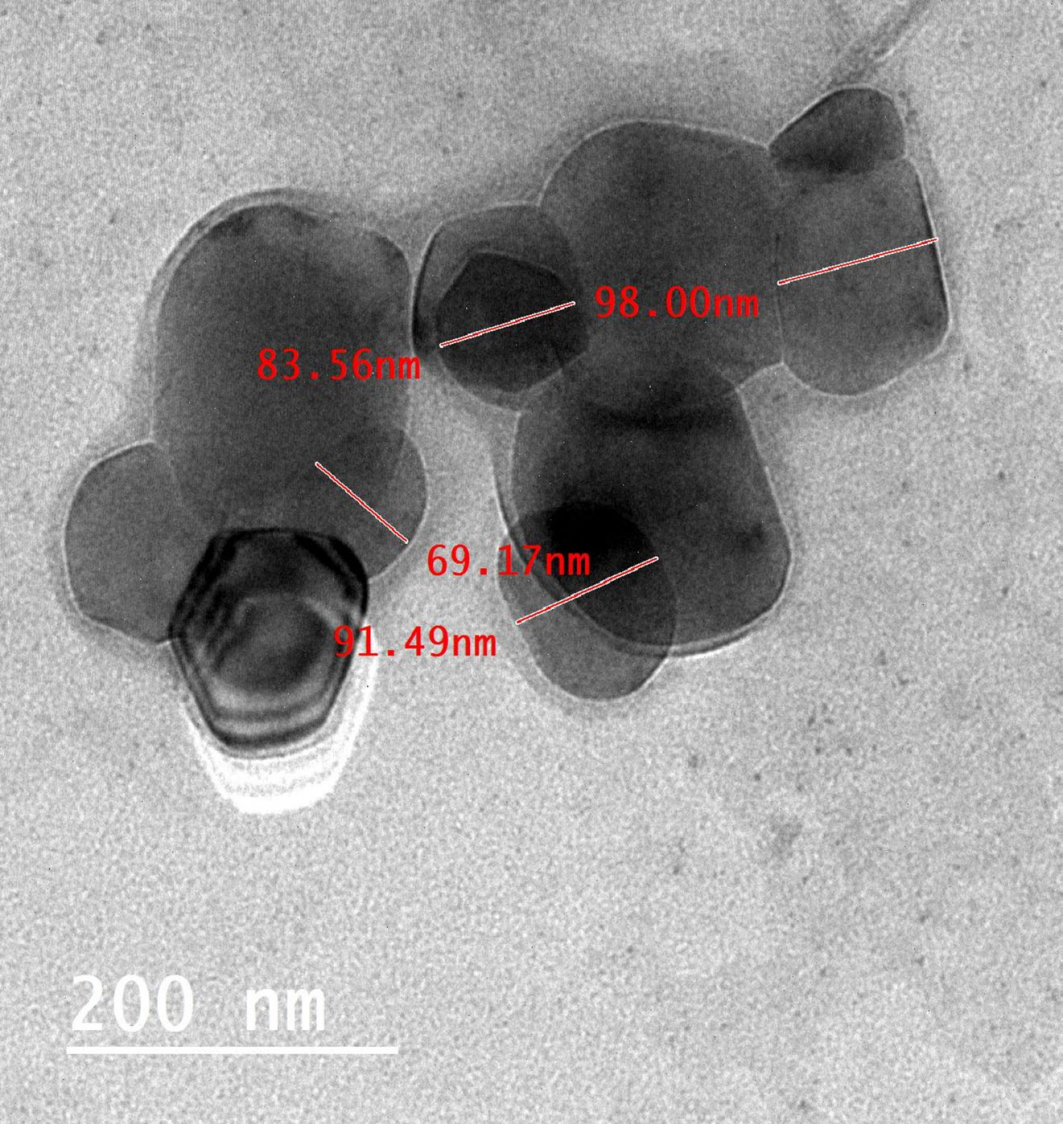
TEM Image of the prepared hyaluronate coated novasomes. TEM image of [BV1]. The image was acquired at 100,000× magnification using a 200 kV accelerating voltage. The scale bar represents 200 nm. The observed vesicles have an average size of ~84 nm.

The morphological structure of the selected formulation (F1) was visualized by TEM (Fig. 4). They were spherical in shape and free from aggregations. The nanovesicles showed very homogenous morphology with a porous surface, including tiny dark-stained dots referring to the extract successfully enclosed in the selected nanosystem cavities, plus a quite uniform particle-size distribution. The particle size diameters obtained by TEM imaging were around 91.49 nm. The average hydrodynamic size (Table 5) determined by the DLS particle size analyser was larger than the TEM images because of the tendency of the nanocoated vesicles to aggregate. TEM images offer lower aggregation of nanoparticles and easier observation of the size and shape of nanoparticles.

**Table 5:** PS, ZP and PDI of the selected hyaluronate-coated novasomes BV1

Formulation	Drug content (%)	PS (nm) ± SD	ZP (mV) ± SD	PDI
BV1	62.03 ± 0.12	340.2±1.58	-34.05 ± 3.28	0.455

#### Fourier-transform infrared spectrometry (FTIR)

**Fig. 5:**
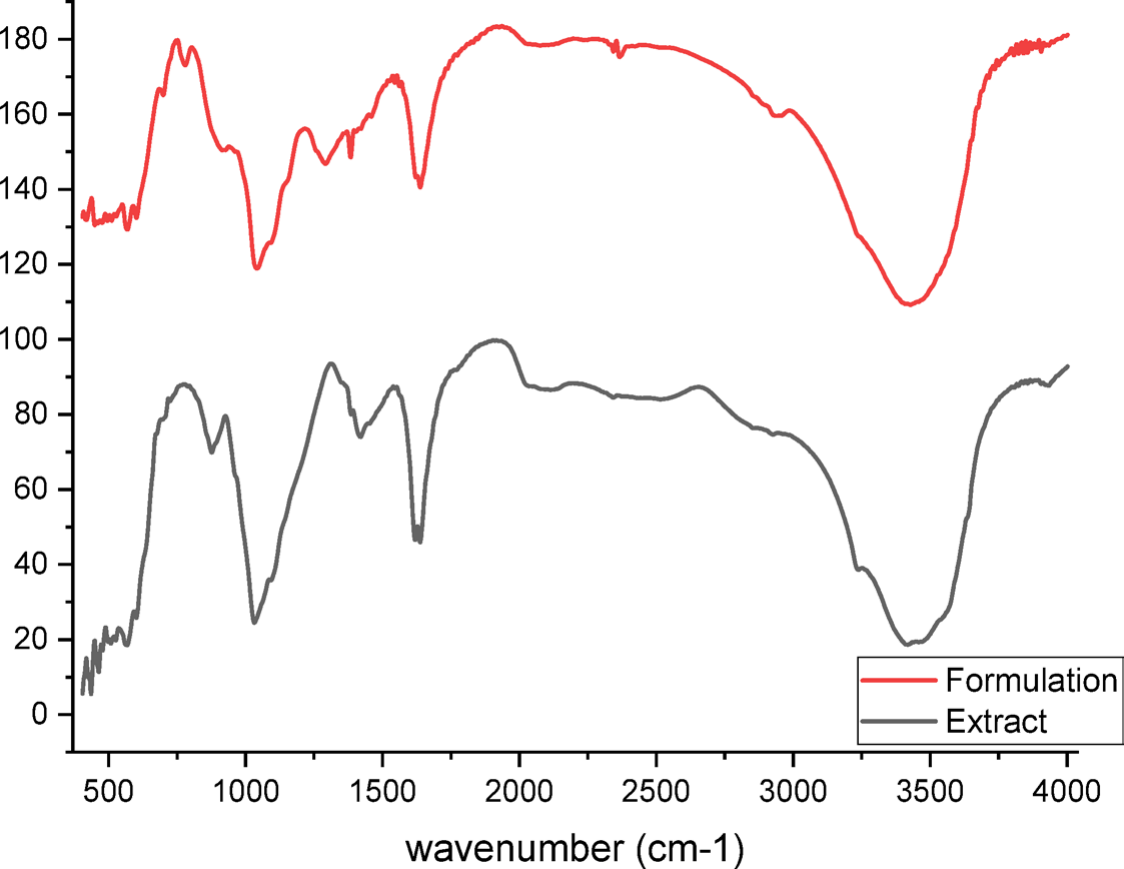
FTIR spectra of the crude extract and the selected prepared hyaluronate coated novasomes

Figure 5 displays the FTIR spectra of extract and extract-loaded formulation F1. The reported characteristic peaks of extract^30^ come in agreement with the present results suggesting the purity of the extract under investigation. The main peaks reported for the extract appear at 3430, 1620, and 1050 cm⁻¹. The peak at 3430 cm⁻¹ corresponds to CH₂ symmetric and asymmetric vibrations, while the peak at 1050 cm⁻¹ corresponds to C-H symmetric and asymmetric bends. The formation of novasomes loaded with extract is confirmed by the FTIR spectra of the formulation BV1 comparing the spectra of the crude pure extract with the spectra of BV1. FTIR spectra showed changes in peak intensity and position of extract after inclusion in BV1. The extract peaks at 3430 cm⁻¹ and 1050 cm⁻¹ are shifted or fused in the IR spectra of the BV1, which indicates interactions between the ingredients and the extract and the successful inclusion of the extract into the vesicles. The successful development of these nano-systems and the beneficial substance encapsulated within them may be demonstrated by the decrease in the band’s intensity of the prepared nanovesicles and the elimination of other bands.

## Discussion

While the zones of inhibition for the novasome formulations were numerically low and did not reach statistical significance (*P* > 0.05), the results indicate a broadening of the antimicrobial spectrum compared to the free extract. The antimicrobial efficacy of the *Beta vulgaris* subsp. *cicla*extract is likely driven by the synergistic action of its identified flavonoids and phenolic compounds, particularly vitexin, kaempferol, and quercetin. Previous research has established that these metabolites exert antibacterial effects by disrupting the integrity of the bacterial cell membrane, inhibiting membrane-bound enzymes, and causing cytoplasmic leakage^31^. The failure of our crude extract to inhibit *E. coli*—a Gram-negative bacterium known for its robust lipopolysaccharide outer membrane—suggests that the bioactive compounds lacked the bioavailability or membrane-penetrating capacity required to reach their intracellular targets. However, the encapsulation of the extract into hyaluronate-coated novasomes (BV1) bridged this gap, conferring moderate activity against both *S. aureus* and *E. coli*. This enhancement is consistent with findings by Mohammed et al., who identified flavone C-glycosides as promising antibacterial agents. The lack of significance at this stage is attributed to the inherent variability of crude extract bioassays, suggesting that further optimization of formulation loading may be required to amplify these inhibitory effects.

The DPPH assay results confirmed that the antioxidant potency of *B. vulgaris*extracts is concentration-dependent. Our findings align with previous reports by Elmastas et al.^32^, and Čeryová et al.^4^, which highlight the influence of extraction solvents and plant parts on phenolic content and subsequent antioxidant efficacy.

Notably, BV1 (IC_50_ 22.48 mg/mL) demonstrated significantly higher antioxidant activity than the crude extract (IC_50_ 30.05 mg/mL). The statistical validation (*P*< 0.05) confirms that this enhancement is a result of the delivery system’s architecture rather than mere concentration effects. The hyaluronate coating likely increases the stability of the antioxidants, preventing degradation before they react with the DPPH radical. Furthermore, as the extract is lipophilic, the nanocarrier system acts as an aqueous-compatible bridge, enhancing the interaction between the antioxidants and the methanol-soluble DPPH radicals^33^^,34^.

The superior performance of BV1 compared to BV2 highlights the crucial role of formulation optimization. The increased concentration of Span 60 in BV1 was pivotal in enhancing the physicochemical properties of the vesicles. Span 60, characterized by its long-saturated acyl chains (palmityl *C*_16_ and stearyl *C*_18_respectively), facilitates the formation of highly stable, less “leaky” bilayers with high phase transition temperatures^34^. This structural stability results in higher drug entrapment efficiency, as the rigid acyl chains prevent the premature release of the encapsulated plant extract. While Span 60 possesses no inherent antioxidant or antimicrobial activity, our data confirms that its role as a surfactant in the novasome system is essential to “potentiate” the activity of the encapsulated actives by improving their dispersion and diffusion into the agar matrix^35^.

## Conclusion

This study successfully demonstrated that the bioactivity of *Beta vulgaris* subsp. *cicla* extract can probably be enhanced through encapsulation in hyaluronate-coated novasomes. While the crude oily extract exhibited limited antimicrobial efficacy, particularly against Gram-negative bacteria, the optimized BV1 formulation showed preliminary inhibitory trends against *S. aureus*,* E. coli*, and *C. albicans*, suggesting a potential expansion of the therapeutic spectrum compared to the crude extract, despite these effects not reaching statistical significance at the tested concentrations. Furthermore, the nanostructured delivery system improved the antioxidant capacity, reducing the IC_50_ to 33.4 µL/mL, a significant improvement over the 55.2 µL/mL observed in the crude form. The superior performance of BV1 may be attributed to the synergistic effect of the hyaluronate coating and the high Span 60 content, which ensured stable, high-capacity encapsulation of lipophilic biomarkers like oleanolic acid and flavonoids. These findings establish hyaluronate-coated novasomes as a superior and novel platform for the delivery of sensitive plant extracts and emphasise the potential of hyaluronate-coated novasomes as an eco-innovative and sustainable strategy for the topical delivery of botanical actives, which are thought to contribute as a promising alternative for the treatment of skin infections and oxidative stress-related disorders.

## Materials and methods

### Phytochemical investigation

#### Plant material and extract preparation

Fresh aerial parts of *Beta vulgaris* subsp. *cicla were* collected from the local market, Giza, Egypt, in January 2023. The identification of the plant material was confirmed by Ms. Therese Labib, botanical specialist and consultant at Orman Botanical Garden. A voucher specimen (M 275) was deposited at the Pharmacognosy Dept., National Research Centre, Cairo, Egypt. The collected aerial parts (3 kg) were air-dried, powdered and kept in tightly-closed containers. The air-dried powder of aerial parts (250 g) of *Beta vulgaris* subsp. *cicla* was then extracted with 4 L ethanol to yield 55.8 g then extracted with dichloromethane to yield 15 g. Each extract was concentrated by using a rotary evaporator, dried and kept in well-closed containers.

#### Chemical evaluation of the dichloromethane fraction using GC-MS

At the Department of Medicinal and Aromatic Plants Research, National Research Centre, and under the subsequent conditions, the dichloromethane fraction was subjected to GC-MS analysis using a gas chromatography-mass spectrometry instrument presented: TG-WAX MS column (30 m x 0.25 mm i.d., 0.25 μm film thickness), coupled with an ISQ Single Quadrupole MS Spectrometer as a detector, and a TRACE GC Ultra Gas Chromatograph (THERMO Scientific Corp., USA). Helium was employed as a carrier gas with a flow rate of 1.0 mL/min using the following temperature program: 60 °C for 1 min, followed by a rise of 4 °C/min to 300 °C and a 15-minute rest. Both the injector and the detector had a temperature of 280 °C. At 70 eV, MS spectral measurements were collected using (EI). The analytical approach of mass spectra (genuine chemicals, Wiley spectral library collection, and NSIT library) was used to recognize the substances.

#### LC-ESI-MS/MS

##### Instrument

The analysis of the sample was performed using liquid chromatography–electrospray ionization–tandem mass spectrometry (LC-ESI-MS/MS) with an Exion LC AC system for separation and SCIEX Triple Quad 5500 + MS/MS system equipped with an electrospray ionization (ESI) for detection in chromatography laboratory at Center of Scientific Excellence, National Research Center.

##### Method of LC-ESI-MS/MS in negative ionization modes

A Ascentis^®^ Express 90 Å C18 Column (2.1 × 150 mm, 2.7 μm) was used for separation. Two eluents, A: 5 mM ammonium formate pH 8 (-ve) and B: acetonitrile (LC grade), made up the mobile phase. 5% B at 0–1 min, 5–100% B from 1 to 20 min, 100% B from 20 to 25 min, 5% at 25.01, and 5% from 25.01 to 30 min were the planned mobile phase gradients. The injection volume was 5 µl, and the flow rate was 0.3 ml/min. Negative ionization mode was used for MS/MS analysis using a scan (EMS-IDA-EPI) for MS1 between 100 and 1000 Da with the following parameters: 25 psi of curtain gas IonSpray voltage: −4500 (-ve); source temperature: 500 °C; ion source gases 1 and 2 were between 50 and 1000 Da for MS2 with a declustering potential of −80; collision energy: −35 (-ve).

##### Isolation and identification of the major compounds

The dichloromethane fraction was loaded on preparative TLC using benzene - ethyl acetate (8:2 v/v). spraying with 10% H_2_SO_4_. The colored bands were marked and gathered. Also, the ethanol extract was loaded on preparative TLC using Dichloromethane: methanol (9:1v/v). The plates were examined under the UV light at 254 nm and 365 nm The Rf values of the isolated compounds were determined. The structure elucidation of some the obtained compounds was confirmed by different spectral analyses (H1-NMR, ^13^C-NMR and MS).

### Preparation of sodium hyaluronate coated novasomes

Oleic acid, span 60 (Sorbitan monostearate) and cholesterol were obtained from sigma Aldrich Co. USA, Sodium hyaluronate (molecular weight 90–100 kDa) was obtained from Shanghai Yuanye Bio-Technology Co., China. Tego care 450 (polyglyceryl-3 methyl glucose distearate) was donated from Goldschmidt (Now Evonik, Essen, Germany). Dichloromethane (HPLC grade) was supplied by Fisher Scientific, UK.With a few adjustments, the solvent injection procedure was used to prepare extract-coated novasomes. To manufacture 0.1% (w/w) extract-loaded novasomes, the extract was dissolved in dichloromethane and injected into a water phase that contained 10 milliliters of distilled water. To the solution of Cholesterol (100 mg), span 60 (SAA; 250, or 500 mg) and oleic acid (FAA;250 mg) in 10 ml dichloromethane, the solution of the extract in water was added drop wise. Then it was left under stirring at 50 °C to evaporate the dichloromethane. 0.5% sodium hyaluronate^36^ was magnetically stirred until fully dissolved. Following that, the produced novasomes after evaporation were magnetically agitated at 1000 rpm while a sodium hyaluronate solution was added. To reduce variance, the container’s and the magnetic bead’s sizes were maintained consistent. For stability, the finished product was agitated for a further half hour.

### Biological investigation methods

#### Antimicrobial activity

Antimicrobial activity of *Beta vulgaris* extract (BV) and its novasomes formulations (BV1, BV2) were tested using agar well diffusion method against *Staphylococcus aureus* ATCC 6538, as an example of Gram-positive bacteria, *Escherichia coli* ATCC 25,922, as an example of Gram-negative bacteria and *Candida albicans* ATCC 10,231, as yeast^37–39^. Tested microorganisms were obtained from the American Type Culture Collection (Manassas, VA, USA) and maintained on Luria-Bertani agar at 4 °C. An inoculum of each organism was prepared and adjusted to a 1.5 × 10^8^ CFU/mL (0.5 McFarland standard) from which (100 µL) were spread on petri dishes containing 20 mL of nutrient agar in case of bacteria or potato dextrose agar in case of yeast^38^. To provide a quantitative dose-response profile, a serial volume-to-volume (v/v) dilution of the neat BV oily extract was prepared in DMSO to achieve concentrations of 1000, 500, 250, and 125 µL/mL. Each well was loaded with 100 µL of the sample. DMSO (100 µL) was used as negative control to ensure the absence of solvent interference, while chloramphenicol and nystatin were used as positive controls for bacteria and yeast respectively. After incubated for 24 h at 37 °C the antimicrobial activity was determined by measuring the diameter of the zone of growth inhibition (ZOI) around the wells in cm^38^. All tests were performed in triplicate, and results are expressed as mean ± standard deviation (SD). Statistical significance was evaluated using one-way ANOVA followed by Tukey’s post-hoc test.

#### Antioxidant activity

DPPH (2,2-diphenyl-1-picrylhydrazyl) radical scavenging assay was employed to test the antioxidant potential of a serial volume-to-volume (v/v) dilutions of crude extract (BV), novasome 1 (BV1) and 2(BV2) in DMSO according to Zhao et al^40^. to obtain concentrations of (100, 50 and 25%). From each dilution, (100µL) was added to (900µL) of (0.1 mM) DPPH solution in methanol resulting in a 10-fold dilution of the sample in the final reaction mixture (final concentrations: 100, 50, and 25 µL/mL), mixed well and left in dark for 30 min at temperature 37 °C. Percent of decolorization of DPPH was determined spectrophotometrically by measuring the reaction mixture absorbance at 517 nm and applying the following equation:

$$\:Scavenging\:activity\:\left(\%\right)=\left(\frac{{A}_{Control\:-}\:{A}_{Sample}}{{A}_{Control}}\right)\times\:100$$

where $\:{A}_{Control}$ is the absorbance of the DPPH radical solution (without sample) and $\:{A}_{Sample}$ is the absorbance of the reaction mixture containing the Beta vulgaris extract or the novasome formulations.

The IC50 value (concentration providing 50% inhibition) was calculated by plotting the percent scavenging activity against the corresponding extract concentrations in the reaction mixture (µL/mL) using linear regression analysis. Calculations were performed using MS Excel.

All assays were performed in triplicate, and results are expressed as mean percentage inhibition ± standard deviation (SD). One formulation was selected based on the antimicrobial and antioxidant activities for further evaluations.

### Characterization of the selected formulation

#### Extract content (%)

One gram, of the extract vesicles was taken in order to determine the encapsulation efficiency (EE)%. Dimethyl sulphoxide (DMSO) was used to dissolve the vesicles finely, and they were then agitated for 24 h using a magnetic stirrer^41^ samples were withdrawn, filtered, diluted suitably and measured spectrophotometrically^42^ for extract content at a wavelength of 745 nm. In summary, 1 milliliter was sampled, followed by the addition of 5 milliliters of diluted Folin Ciocalteu Reagent (Folin/water 1:9 v/v) and (4 mL) of 7.5% sodium carbonate (w/v). After a 24-hour dark incubation phase, the mixture was mixed with a vortex mixing and examined^43^.The EE% was defined as the ratio of the quantity of extract entrapped in these generated vesicular systems to the initial extract added content of the formulation. All experiments were performed in triplicates. Encapsulation efficiency (EE) % was calculated using the Eq^44^.:

$$\:\mathrm{E}\mathrm{E}\mathrm{\%}=\frac{\mathrm{q}\mathrm{u}\mathrm{a}\mathrm{n}\mathrm{t}\mathrm{i}\mathrm{t}\mathrm{y}\:\mathrm{o}\mathrm{f}\:\mathrm{e}\mathrm{x}\mathrm{t}\mathrm{r}\mathrm{a}\mathrm{c}\mathrm{t}\:\mathrm{e}\mathrm{n}\mathrm{t}\mathrm{r}\mathrm{a}\mathrm{p}\mathrm{p}\mathrm{e}\mathrm{d}}{\mathrm{T}\mathrm{o}\mathrm{t}\mathrm{a}\mathrm{l}\:\mathrm{q}\mathrm{u}\mathrm{a}\mathrm{n}\mathrm{t}\mathrm{i}\mathrm{t}\mathrm{y}\:\mathrm{o}\mathrm{f}\:\mathrm{e}\mathrm{x}\mathrm{t}\mathrm{r}\mathrm{a}\mathrm{c}\mathrm{t}\:\mathrm{a}\mathrm{d}\mathrm{d}\mathrm{e}\mathrm{d}\:}\mathrm{X}100$$

#### Particle size, PDI and zeta potential measurements

Particle size, PDI, and zeta potential (ZP) were evaluated using photon correlation spectrometry using the Malvern Zetasizer Nano ZS. The dispersion was doubly diluted with room temperature distilled water and then briefly sonicated in a bath before measurement. Before the sample was put in a quartz cuvette for room-temperature analysis, one milliliter of each vesicular system was appropriately diluted with up to ten milliliters of double-distilled water^45,46^.

#### Transmission electron microscopy

The morphological characteristics of the selected vesicular system were assessed in order to quantify structural aspects, such as size and shape homogeneity. The selected formulation was diluted with 1:10 bi-distilled water just prior to the investigation. Each sample was placed on a carbon-coated grid with a mesh size of 300 and left to air dry at room temperature. Before the film had completely cured, 1% phosphotungestic acid severely stained it. A TEM (JEOL, JEM-1230, Tokyo, Japan) operating at room temperature and 80 KV of acceleration was used to evaluate the film before micrographs were taken at the proper magnifications.

#### FTIR

An FT-IR spectrophotometer (JASCO 6100, Tokyo, Japan) was used to perform spectral analyses of the extract and the selected lyophilized system in order to identify any potential interactions between the formulation components. Potassium bromide (KBr) and solid samples (the extract and the freeze-dried sample) were combined separately and compressed for two minutes at 200 kg/cm^2^ pressure to create compact discs. All samples were scanned between 4000 and 400 cm-1 Hz against a blank KBr pellet background^46^.

## Data Availability

All data generated or analyzed during this study are included in this article and its supplementary information file.

## Abbreviations

BV: *Beta vulgaris* subsp. *cicla*
BV1: Formula 1
BV2: Formula 2
FTIR: Fourier-transform infrared spectroscopy
TEM: Transmission electron microscopy
GC/MS: Gas chromatography–mass spectrometry
LC–MS/MS: Liquid chromatography–tandem mass spectrometry
DPPH: 2,2-diphenyl-1-picrylhydrazyl
